# The Olden Time

**Published:** 1860-05

**Authors:** 


### THE OLDEN TIME.

"WHAT are you doing there, Pat? digging a hole?"

"No, your honor; I'm digging out the dirt, and leaving the hole there!"

In the early times of the old "Chamberlain," we were elected to the office of "critic," and kept it all the days of our sojourn at college, with short exceptions, to go up higher. We were so full of fun, and so merciless on delinquencies, that if at any part of the "session" there was "a full house," it was sure to be when "Father Hall" had his reports to make. How well we remember being a committee of one about some trifling repairs to be made, costing perhaps ten cents, and presenting a report of ever so many pages. Such uproarous cachinnations never came so near raising the roof of "Old Center." And only think of it! The society, by a unanimous vote, ordered it to be read a second time, and then to be placed in full on the minutes, when such men were there as Gideon Blackburn, and David Nelson, author of that unsurpassable book, "The Cause and Cure of Infidelity," and John Green, and Nathan L. Rice, and others who have since become eminent in their day and generation, such as Lewis W. Green and—ahem!—the Editor of HALL'S JOURNAL OF HEALTH, etc., and so on.

About this time it was our custom now and then to go out to "Judge Green's," where our correspondent lived. We asked him a very simple question, and with his remarkable vivacity, promptness, and fearlessness, he replied thereto.

"What is a hole, C.?"

"It's an orifice bounded by space."

"If in a pane of glass, does space bound it?"

"Yes, at the two ends."

"But the side boundaries?"

"Then a hole has two ends and a side."

"But suppose it's a well?"

"Then all the end is in one direction!"

"Won't do, C., try it again. What is a hole?"

"It's a vacancy surrounded by circumstances, varying according to the nature of the case."

There's fun in the remembrance of these things, as there was fun in the action of them. Blessed times they! when all was sunshine, emblematic of the better country on the thither side of Jordan, where we shall be always young, always happy, always good. Surely it is worth a lifetime of special effort to reach that land. Another "thirty," and we'll both be there, to go no more out. Reader, may you be along!

Such were the thoughts suggested on receiving the following from an old college-mate, who has made himself a place and a name among men, and whose work has been neither wood, hay nor stubble, but "goodly stones" in the great building:

"I READ IT STRAIGHT THROUGH!

"You ask, 'What?' HALL'S JOURNAL for March. I began just after closing my Greek Testament in the morning, intending five minutes for glancing over its contents. Despite of the weightier matters pressing, an hour was gone, and I was at the end of a number which no one can read without profit. That loss of yours! What use had pickpockets for your manuscript? If they will pay good prices, I can furnish them some from the same hand, written in other days and under other skies. I wonder if you would know yourself were I to report to you a bundle of extracts? Is it the same *Hall* that I used to see and hear in the academic shades of Old Center? that sat at the same table at Aunt Tabitha's? that met us in the intellectual gymnastics of the *Chamberlain Hall above the chapel?* that wrote me those valued epistles when our paths diverged so widely? I see very clearly the ear-marks of mental identity. But the physical temple has been four times taken down and re-built. Has this process been like the re-building in your metropolis-home—for a cottage, a palace; for a city of brick, a city of marble? Then I must look out for a *sturdy athlete—the six-foot stature,* and the *raven black hair* gracefully falling over that '*teeming dome of thought* and palace of the soul.'

"I can not find words to express my high appreciation of the moral animus of your JOURNAL. *The right and the true forever!* This I perceive to be your motto, though no where blazoned in golden letters. I was present at the sowing-time; the harvest has for years *been ripening.* We see how the ancient law abides —the seed yields after its kind. '*Let the earth bring forth grass, the herb yielding seed, and the fruit-tree yielding fruit after his kind.*'

I see how the same law sweeps up into the higher ranges of the moral world. Once we did meet at Irving Place. You marked it by your able volume on throat diseases, placed in my hands for old acquaintance' sake. Before I got home I met a clergyman of distinction, sinking rapidly. His charge was surrendered; great ulcers were choking his utterance. I lent him the book. All that followed I do not know. This much I can testify: *that minister became a successor of the late and revered Dr. Spencer, and now is Professor in the great N. W. Theological Seminary.* He started the book to me in my Ohio home, through the Post-Office. I suppose some suffering official intercepted it, as it never reached me. H. G. C."

